# Controllable vortex lasing arrays in a geometrically frustrated exciton–polariton lattice at room temperature

**DOI:** 10.1093/nsr/nwac096

**Published:** 2022-05-14

**Authors:** Jun Wang, Yutian Peng, Huawen Xu, Jiangang Feng, Yuqing Huang, Jinqi Wu, Timothy C H Liew, Qihua Xiong

**Affiliations:** Division of Physics and Applied Physics, School of Physical and Mathematical Sciences, Nanyang Technological University, Singapore 637371, Singapore; Department of Optical Science and Engineering, and Shanghai Frontiers Science Research Base of Intelligent Optoelectronics and Perception, Fudan University, Shanghai 200433, China; State Key Laboratory of Low-Dimensional Quantum Physics and Department of Physics, Tsinghua University, Beijing 100084, China; Division of Physics and Applied Physics, School of Physical and Mathematical Sciences, Nanyang Technological University, Singapore 637371, Singapore; Division of Physics and Applied Physics, School of Physical and Mathematical Sciences, Nanyang Technological University, Singapore 637371, Singapore; Division of Physics and Applied Physics, School of Physical and Mathematical Sciences, Nanyang Technological University, Singapore 637371, Singapore; Division of Physics and Applied Physics, School of Physical and Mathematical Sciences, Nanyang Technological University, Singapore 637371, Singapore; Division of Physics and Applied Physics, School of Physical and Mathematical Sciences, Nanyang Technological University, Singapore 637371, Singapore; State Key Laboratory of Low-Dimensional Quantum Physics and Department of Physics, Tsinghua University, Beijing 100084, China; Beijing Academy of Quantum Information Sciences, Beijing 100193, China

**Keywords:** vortex lasing, exciton–polariton lattice, Bose–Einstein condensation, the orbital angular momentum of light, geometric frustration, perovskite semiconductor

## Abstract

Quantized vortices appearing in topological excitations of quantum phase transition play a pivotal role in strongly correlated physics involving the underlying confluence of superfluids, Bose–Einstein condensates and superconductors. Exciton polaritons as bosonic quasiparticles have enabled studies of non-equilibrium quantum gases and superfluidity. Exciton–polariton condensates in artificial lattices intuitively emulate energy-band structures and quantum many-body effects of condensed matter, underpinning constructing vortex lattices and controlling quantum fluidic circuits. Here, we harness exciton–polariton quantum fluids of light in a frustrated kagome lattice based on robust metal–halide perovskite microcavities, to demonstrate vortex lasing arrays and modulate their configurations at room temperature. Tomographic energy–momentum spectra unambiguously reveal massless Dirac bands and quenched kinetic-energy flat bands coexisting in kagome lattices, where polariton condensates exhibit prototypical honeycomb and kagome spatial patterns. Spatial coherence investigations illustrate two types of phase textures of polariton condensates carrying ordered quantized-vortex arrays and π-phase shifts, which could be selected when needed using lasing emission energy. Our findings offer a promising platform on which it is possible to study quantum-fluid correlations in complex polaritonic lattices and highlight feasible applications of structured light.

## Teaser

Room-temperature Bose-Einstein condensation and quantized-vortex arrays are realized and manipulated in a polaritonic kagome lattice, which provides a promising optical analogue for emulating the formation of flat bands and arrangements of vortical spin.

## INTRODUCTION

Geometric frustration is an intriguing characteristic in condensed matter physics [1], stemming from an inaccessible minimum-energy ground state with a global ordered arrangement of spins in the system [2]. A 2D kagome lattice, a pattern of corner-sharing triangular plaquettes, is a representative system with a particularly high degree of frustration, which provides a fertile ground to explore the interplay between spin, orbital and non-linear phenomena in magnetics and topological systems, based on two vital features of both multiple attainable spin configurations and band structures including flat bands and Dirac cones. The frustrated lattices accompanied by quantum destructive interference bolster non-dispersing flat bands [3]. By modulating Chern numbers of Dirac bands for kagome lattices, non-trivial topological phases have been achieved in various systems involving photonic waveguides [4], photonic crystals [5] and acoustics [6]. On the other hand, the vortical spin configurations, derived from a competition between intrinsic interactions and the lattice geometry, endow frustrated lattices with many-body effects, such as itinerant–electron ferromagnetism [7], the giant anomalous Hall effect [8], spin ices and spin glasses [9].

Quantized vortices are one of the intriguing quantum effects, carrying quantized phase winding and the circulation of superfluids around a phase singularity, which is fundamental to the studies of topological excitations [10] and Kosterlitz–Thouless transitions [11] in interacting Bose gases. Exciton–polariton condensates, resulting from non-equilibrium Bose–Einstein condensation of excitons hybridized with confined photons, are deemed to be out-of-equilibrium quantum fluids of light [12], underpinning macroscopic quantum phenomena represented by quantized vortices [13–16], superfluidity [17] and Bogoliubov excitation [18]. Extra geometrical structures are able to induce ordered quantized vortices, which have been demonstrated by ultracold atoms in an optical lattice [19]. Considerable demonstrations and dynamics of quantized vortices in quantum fluids are realized at cryogenic temperature [20,21] and their configurations could be pinned in random topological defects or tunable optical potentials [22–26]. Therefore, here remain challenges in implementing bosonic vortex lattices with microstructural modulation at room temperature. Harnessing artificial periodic potential landscapes [27,28] imposed on the photonic component of polaritons, so-called polaritonic lattices intuitively demonstrate energy-band structures and exotic quantum behavior of condensed matter involving topological insulators [29,30], classical spin simulators [31] and gap solitons [32]. Combining boson attributes with artificially frustrated potentials, the polaritonic kagome lattice can demonstrate a clear bosonic condensation in the metal-film deposition microcavity system at a cryogenic temperature [33]. However, few experimental studies have so far addressed generating room-temperature ordered vortex lattices and modulating their configurations when needed via various reconstructed spatial arrangements of polariton condensates in kagome lattices. Such vortex lattices assisted by the imposed geometry could describe an optical analog for emulating the versatile spin and orbital physics of spin systems [34–36]. The spatially individual topological charges carried by such vortex lasing provide an opportunity to expand the additional degree of freedom of light emission and propagation in solid-state photonic and optoelectronic devices.

In this article, we demonstrate the kagome Hamiltonian in a 2D polaritonic lattice at room temperature, based on robust metal–halide perovskite semiconductors embedded in microcavities. The full energy-band structure of kagome lattices, hosting massless Dirac cones and infinite-mass flat bands, are unambiguously revealed by tomographic energy–momentum spectra and theoretical calculations. In the non-linear regime, thanks to the driven-dissipative nature of polaritons, a considerable number of polaritons simultaneously condense at two pivotal states of the lattice, i.e. Dirac points (DPs) and the flat band. Relying on geometric frustration of the lattice, ordered (anti-)vortex lasing and non-vortical condensates have simultaneously been achieved, where the spatial patterns and topological charges can be optically selected by lasing emission energies. Our results propose a promising platform for emulating how geometric frustration affects arrangements of quantized vortices to form flat bands and actualizing topologically protected light sources and devices.

## RESULTS AND DISCUSSION

### Schemes and fabrications of the polaritonic kagome lattice

**Figure 1.**
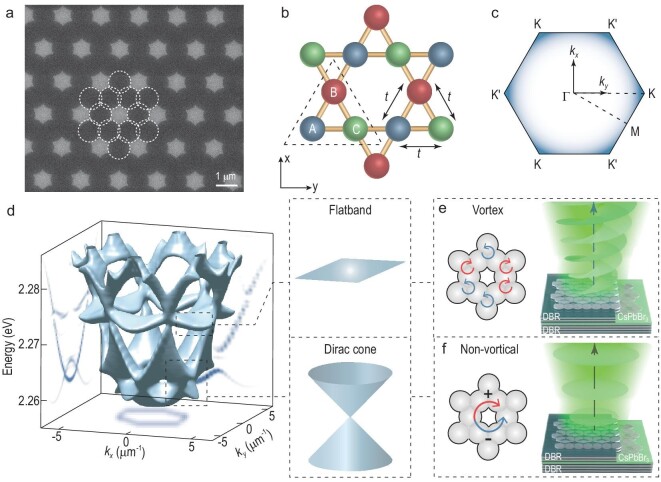
Schematics of exciton–polariton vortices in the perovskite kagome lattice. (a) An image of scanning electron microscopy of a 2D kagome lattice on the perovskite layer before the deposition of the top DBR. The white dashed circles depict the contours of sites. Scale bar: 1 μm. (b) Illustration of a single plaquette of the kagome lattice. The dashed triangle shows a unit cell. The coupling strength *t* between sites is highlighted. (c) Schematic of the first Brillouin zone. (d) Theoretically calculated full band structure. The projections of the $E - {k_y}$, $E - {k_x}$ and ${k_x} - {k_y}$ planes correspond to the cross sections of ${k_x} = - 2.39{\rm{\,\,\mu }}{{\rm{m}}^{ - 1}}$, ${k_y} = 2.77{\rm{\,\,\mu }}{{\rm{m}}^{ - 1}}$ and the first Brillouin zone, respectively. Right panel: schematic zoom-in views of band structures for the flat band and Dirac cone. (e) and (f) Schematics of phase textures of the flat band and DP eigenstates. The vortex–antivortex arrays (vortex lasing) in the flat band and π phase shifts (non-vortical lasing) in DPs are presented.

Our 2D polariton kagome lattice consists of identical coupled micropillars with a diameter of 1 μm and a center-to-center distance of 0.85 μm (Fig. 1a), which is created by patterning the spacer layer of poly(methyl methacrylate) (PMMA) inside a cesium lead bromide (CsPbBr_3_) perovskite planar microcavity that underpins robust room-temperature polaritonic condensation, propagation and lattices [37–39]. The details of polaritons in a single micropillar are described in the Supplementary Materials. The unit cell of kagome lattices is composed of three sites (A, B, C) linked by a nearest-neighbor coupling *t*, schematically shown in Fig. 1b. When considering the strong exciton–photon coupling, a generalized Gross–Pitaevskii (GP) equation is applied to further understand polaritonic kagome lattices, described in more detail in the ‘Methods’ section. Figure 1d shows a theoretically calculated 3D band structure of a polaritonic kagome lattice, characterized by the sets of Dirac bands capped by a dispersionless flat band, in analogy to the prototypical electronic kagome bands [3]. The flat band arising from the anti-bonding of *s* orbitals leads to frustrated phases and equal eigenenergy on each site. The triangular geometry of kagome unit cells can break the initial phase arrangement, reconstructing a steady-state distribution with vortical phases surrounding neighboring sites (Fig. 1e). Utilizing the geometry to create opposite optical orbital angular momentum (OAM) on anti-bonding modes of *s* orbitals, ordered vortex–antivortex lattices are introduced in polaritonic phase textures of the flat band; otherwise, the polaritons in DP eigenstates present a π phase shift with zero vorticity, as schematically shown in Fig. 1e and f.

### Band structures and condensation in the polaritonic kagome lattice

**Figure 2.**
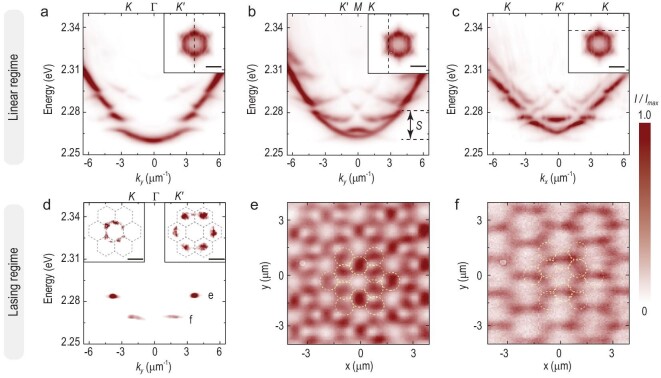
The optical characterization of the polaritonic kagome lattice in the linear and lasing regime. (a)–(c) Energy-resolved momentum-space polariton dispersions of the perovskite kagome lattice along three special directions in momentum space below the critical threshold. The inset is the first BZ in the momentum space, scale bar: 3 μm^–1^. (a): along $K - \Gamma - K^{\prime}$ direction; (b): along $K^{\prime} - M - K$ direction; (c): parallel to ${k_x}$ axis and through *K^ ′^* point. Dirac cones and the flat band are represented in the dispersions. (d) Energy-resolved momentum-space polariton dispersion (along $K - \Gamma - K^{\prime}$ direction) in the lasing regime at the pump fluence of 2.5 *P_th_*, showing two sets of macroscopic occupations of polaritons in DPs and the flat band of the *s* band, respectively. The left (right) inset presents the momentum-space image of the polariton condensates in DPs (flat band) at 2.2692 eV (2.2844 eV), scale bar: 3 μm^–1^. (e) and (f) Energy-selected spatial images of condensate emission at 2.5 *P_th_*, corresponding to two modes in panel (d). Yellow dashed lines depict the contours of sites in the kagome lattice. Exciton polaritons in the flat band condense at the center of each site, exhibiting a typical kagome pattern. Polaritons in DPs condense at the center of three pillars in the unit cell, exhibiting a honeycomb pattern.

To experimentally characterize the full band structures and spatial images of our polaritonic kagome lattice in linear and non-linear regimes, tomographic energy-resolved energy–momentum and real-space photoluminescence (PL) spectra are performed at room temperature. In the linear regime, the lattice is non-resonantly excited by a continuous-wave laser (457 nm) with a Gaussian-shaped spot and weak pumping power. In Fig. 2a–c, kagome band structures are comprehensively characterized with energy–momentum dispersions measured along three special cross sections of momentum space, including the $K - \Gamma - K^{\prime}$direction, the $K^{\prime} - M - K$ direction and parallel to the ${k_x}$ axis through the *K^ ′^* point, corresponding to the dashed line in each inset. The inset to Fig. 2a exhibits the momentum-space polariton emission at the DP energy of the *s* band (2.2656 eV). Six DPs are observed at the corner of the first Brillouin zone (BZ). More experimental and theoretical dispersions scanned along other sections and tomographic momentum-space images are shown in the Supplementary Materials for comparison. The kagome dispersion contains two groups of bands (*s* and *p* bands), separated by an energy gap of ${\rm{\Delta }}E = 5.8$ meV at ${k_y} = \pm 4.16\,\,{\rm{\mu }}{{\rm{m}}^{ - 1}}$ shown in Fig. 2b. The three lowest bands (*s* bands), arising from the coupling between the ground state of the pillars, contain one flat band and two Dirac bands possessing a six-fold rotational symmetry. Figure 2b and c shows two and one typical Dirac linear intersections in the two lowest bands, respectively. Here the third band at the bottom is flat and dispersionless at most momenta due to the destructive interference on the nearest neighbors. At higher energies of >2.2836 eV, the coupling of the first-excited-state polaritons on pillars gives rise to *p* bands; more Dirac cones and negative-mass modes in high-order BZs can therefore be observed.

One of the intriguing features for exciton polaritons is non-equilibrium Bose–Einstein condensation at room temperatures, accompanied by lasing emission. Above the critical threshold (2.5*P_th_*) pumped by a femtosecond laser (see ‘Methods’ section), thanks to the driven-dissipative nature of polaritons, polaritons tend to simultaneously condense at two selected states of *s* bands with maximal gains (Fig. 2d). The insets to Fig. 2d depict the energy-resolved momentum-space images of two such macroscopic-occupation states that are selected using laser-line filters. Combined dispersions with momentum-space PL emissions prove that the lower-energy (2.2692 eV) and higher-energy (2.2844 eV) states correspond to six first-BZ DPs and the flat band, respectively, where polaritons condense at *K* (*K^ ′^*) points (corners of the first BZ) and *Γ*_2_ points, respectively. Momentum-space diffraction patterns with six sharp peaks and high symmetry suggest an extended long-range spatial coherence build-up in the lattice. Figure 2e and f shows the related energy-resolved spatial emission patterns of the flat band and DPs, respectively, corresponding to the two lasing modes in Fig. 2d. The eigenfunctions of polariton condensates in the *s* flat band are extended over the entire structure with emission lobes centered on each micropillar, arranged in a kagome geometry and referred to as a compact localized state (CLS) (Fig. 2e). The CLS is characterized by an infinite effective mass and a suppressed kinetic energy, implying no interaction or weak interactions between neighboring-site polaritons. In our lattice, the overlap of neighboring pillars allows a large hopping strength resulting in the next-nearest-neighbor coupling or mode hybridization, therefore inducing a residual dispersiveness into the flat band. On *s*-band DPs (Fig. 2f), polaritons condense at the center of three merging pillars in the unit cell, due to the formation of bonding modes for these three pillars, where polariton condensates arranged in a hexagonal geometry present analogous attributes to graphene.

### Ordered vortex lasing array in the flat band of the polaritonic kagome lattice

**Figure 3.**
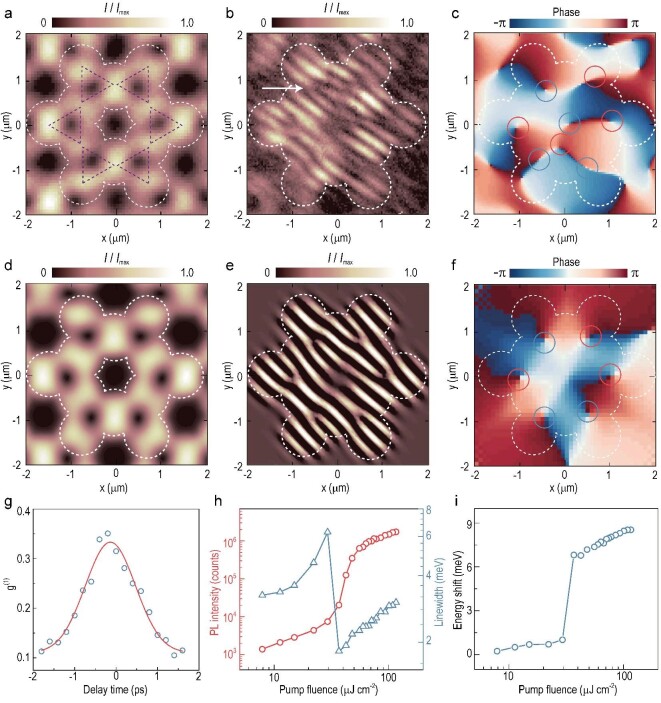
Demonstration of ordered quantized vortices in the flat band. (a) Spatial image of the polariton condensate emission in the flat band. White dashed lines depict the contour of the lattice. The emission density profiles drop to zero at the center of the vortex core. (b) Interferogram of polariton condensates in the flat band. The pitchfork (white arrow) in interference fringes indicates the existence of a quantized antivortex. (c) Phase map extracted from the interferogram of panel (b). Red (blue) circles represent vortices (antivortices) with topological charges of 1 (–1), arranged in an antiparallel order. (d)–(f) Theoretically calculated spatial image, interferogram and phase map, corresponding to panels (a)–(c). (g) Visibility of the interference as a function of the time delay, extracted from panel (b). The red line shows a Gaussian fitting used to extract the coherence time *τ*, where ${\tau _{{\it CLS}}}{\rm{\,\,}}\sim{\rm{\,\,}}1.42$ ps. (h) Integrated intensity (red) and linewidth (blue) as a function of the incident pump fluence, showing the critical threshold of 29.2 μJ cm^–2^ for the flat-band mode of the *s* band, demonstrating a superlinear increase trend and a marked collapse at the threshold. (i) Evolution of the energy blue shift as a function of the pump fluence.

After achieving polariton condensation in the frustrated kagome lattice, we further studied the phase distribution of polariton condensate states via the spatial collective coherence of polariton lasing emission. A non-resonant pulsed excitation laser with linear polarization triggers polariton condensation in the lattice, while the lasing emission is collected using a laser-line filter and a Mach-Zehnder (MZ) interferometer (refer to Supplementary Fig. S4 for more information). We interfere the full emission beam of selected CLS condensates (Fig. 3a) with a magnified image of one lobe from the condensates, which covers a complete kagome pattern and acts as a phase reference (Supplementary Fig. S5). Figure 3b shows the interferogram of CLS condensates. The pitchforks in interference fringes stably sit at the center of each triangular plaquette in the kagome lattice, signifying the emergence of the vortices at these positions. Figure 3c exhibits the corresponding phase mapping translated using an off-diagonal Fourier filtering technique, in which the phases of $\pm 2\pi \,\,$wind around singularities locked at the positions of pitchforks in Fig. 3b. The odd merging pillars in each unit cell of the kagome lattice constitute a closed waveguide loop with the parity symmetry breaking (dashed triangle in Fig. 3a), which could generate phase windings and quantized vortices. Owing to the frustrated geometry and non-resonant excitation with zero-flux, six closed loops of the single kagome plaquette could trap the vortices with opposite topological charges of $l = \pm 1$, forming possible steady-state hexagonal arrangements with a net topological charge of zero (described on in more detail in Supplementary Fig. S7). The vortices and antivortices in our lattice (red and blue circles in Fig. 3c) tend to arrange in an antiparallel-ordered configuration, which is in analogy to one antiferromagnetic mode of the spin system [40]. The theoretically calculated spatial image, interferogram and phase of CLS condensates are in agreement with experimental results, as shown in Fig. 3d–f. The vortex arrangements in the phase texture are dependent on driven-dissipative features and stochastic initial conditions of the system (Supplementary Fig. S7), thus disorder and fluctuations could result in shift, flipping and breakdown of vortices. The phenomenon of quantized vortices is crucial because ordered vortex–antivortex arrays intuitively demonstrate the interplay between polaritonic phases and the frustrated geometry, which intimately connects to the formation of flat bands and the quench of quasiparticle kinetic energy.

More evidence of lasing can be obtained from the polariton coherence time measurement. By moving the mirror of one arm of the MZ interferometer, the delay between the original spatial images and the magnified images can be accurately tuned. Figure 3g shows the first-order correlation ${g^{( 1 )}}( \tau )$ as a function of the delay *τ*, which was extracted from the fringe visibility of a line spectrum across stripes and pitchforks at the pumping fluence of 2.5 *P_th_*. Here, the fringe visibility is defined as ${g^{( 1 )}}\,\,( \tau ) = \frac{{{I_{max}}( \tau ) - {I_{min}}( \tau )}}{{{I_{max}}( \tau ) + {I_{min}}( \tau )}}\,\,$, where ${I_{{\rm{max}}( {min} )}}( \tau )$ is the maximum (minimum) intensity of the envelope function at a delay *τ*. The coherence time of the vortex lasing mode of 1.42 ps is extracted from Fig. 3g, illustrating the localization of polaritons. As polaritons originating from adjacent sites will interact destructively on their shared nearest-neighbor site, the propagation is inhibited, leading to a slightly longer coherence time and suppressed kinetic energy of the flat band, which is an efficient means to implement highly coherent polariton vortex lasers. To characterize the emergence of polariton condensation quantitatively, we demonstrate the evolution of emission intensity, linewidth and peak energy of the CLS condensate as functions of pump fluence. Beyond the critical threshold (29.2 μJ cm^–2^), macroscopic populations of polaritons begin to accumulate into the flat band, as evidenced by a superlinear increase in the emission intensity and correlative narrowing in the linewidth, which symbolizes increased temporal coherence (Fig. 3h). In the meantime, the energy of the polariton emission displays a clear continuous blue-shift trend with the increase in pump fluence (Fig. 3i), mainly stemming from the repulsive polariton–exciton reservoir interactions.

### A polariton lasing with non-vortical phase in DPs

**Figure 4.**
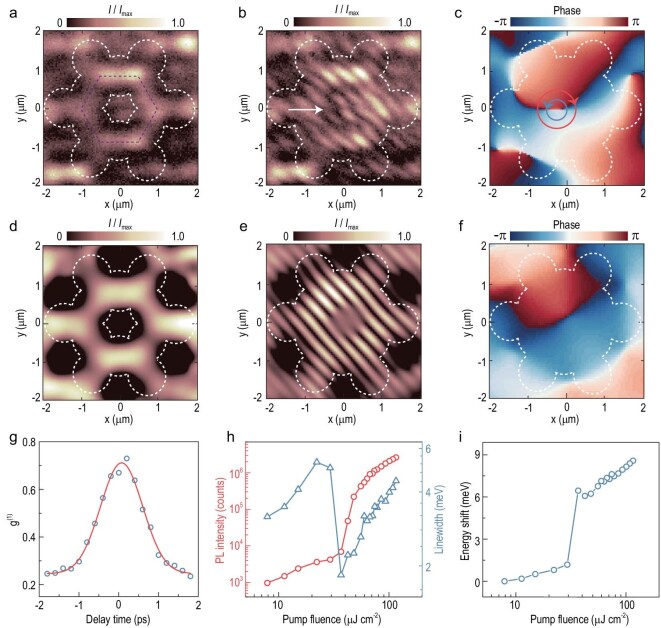
Demonstration of a polariton lasing with non-vorticity in Dirac points. (a) Spatial image of the polariton condensate emission in DPs. White dashed lines depict the contour of the lattice. (b) Interferogram of polariton condensates in DPs. The white arrow points out the existence of staggered fringes. (c) Phase map extracted from the interferogram of panel (b). Polariton condensates in the DP mode present a vortex–antivortex coherent superposition carrying the net OAM of zero, along with a π phase shift in the center of the lattice. (d)–(f) Theoretically calculated spatial image, interferogram and phase map, corresponding to panels (a)–(c). (g) Visibility of the interference as a function of the time delay, extracted from panel (b). The red line shows a Gaussian fitting used to extract the coherence time *τ*, where ${\tau _{DP}}\sim 1.25$ ps. (h) Integrated intensity (red) and linewidth (blue) as a function of the incident pump fluence, showing the critical threshold *P*_th_ ∼ 29.3 μJ cm^–2^ for the DP mode of the *s* band, demonstrating a superlinear increase trend and a marked collapse at the threshold. (i) Evolution of the energy blue shift as a function of the pump fluence.

For the *s*-band DP state, a similar condensation process can be observed, including emission intensity, linewidth and blue shift, but the polariton condensates carry a non-vortical phase in the lattice, i.e. a vortex–antivortex coherent superposition, akin to the scenario in a polaritonic graphene lattice [34,38]. The spatial image of the polariton condensate emission in the DP state hosts a hexagonal pattern with parity symmetry (Fig. 4a). The corresponding interferogram (Fig. 4b) shows discontinuous and staggered fringes between the upper and lower parts, indicating the emergence of a π phase shift in the DP state. The extracted phase (Fig. 4c) verifies that those relative π phase shifts are indeed locked at the center of the hexagonal emission pattern, suggesting the formation of a vortex–antivortex coherent superposition. The polariton emission of a DP state carries a net OAM of zero, due to a superposition with opposite equal-probability OAMs ($l\,\, = \,\, \pm 1$). The phase of such (anti-)vortex winds from 0 to $( - ) + 2\pi $ around closed-loop lobes of condensates. Figure 4c shows that the relative phases between such opposite vortices are shifted by integer multiples of π in two parts inside the dashed envelope, leading to a maximal constructive interference. Such phase ordering and build-up of spatial coherence in our lattice are theoretically simulated by the GP equation, as shown in Fig. 4d–f. The coherence time of 1.25 ps extracted from the visibility of the DP state is shorter than that of the flat band (Fig. 4g). The evolution of emission intensity, linewidth and peak energy of the DP state show that the threshold of 29.3 μJ cm^–2^ is comparable to that of the CLS (Fig. 4h and i). Harnessing the gain–loss mechanism of non-equilibrium condensation, we could modulate the polaritons to condense at the specific states, which carry different vortex configurations, including ordered vortex–antivortex arrays and π phase shifts, via tuning the cavity detuning. Our scheme assisted by an exceptional geometric-frustration structure provides a feasible approach to enhancing the coherence of polariton lasers and generating spatially separated OAM modes of light.

## CONCLUSION

We have realized a 2D exciton–polariton kagome lattice in perovskite microcavities at room temperature, unambiguously revealing the full dispersions with Dirac bands and flat bands. We demonstrated that polaritons simultaneously condense at DPs and the geometrically frustrated flat band, which hold typical honeycomb and kagome spatial patterns, respectively. By employing artificial periodic potential landscapes, we have realized ordered vortex–antivortex lattices and π phase shifts in polaritonic phase textures. The emergence of vortex–antivortex lattices of flat-band condensates manifests the effects of many-body interactions in geometric frustration and quenched kinetic energy. Furthermore, such a phase texture permits our polariton vortex lasing with active tunability of spatially topological charges, which could be selected using an energy filter. Our work reveals an alternative avenue for studying interacting quantum fluids of light in the flat-band system and implementing high-order topological polariton lasers and vortex switches operating at room temperature.

## METHODS

### Perovskite lattice fabrication

In total, 20.5 pairs of titanium oxide and silicon dioxide were deposited using an electron-beam evaporator as the bottom distributed Bragg reflector (DBR). The CsPbBr_3_ perovskite single crystal was grown on a mica substrate using a vapor phase deposition and transferred onto the bottom DBR using a dry-transfer method with cellophane tape. The exciton energy of CsPbBr_3_ is 2.406 eV. A 60-nm-thick PMMA spacer was spin coated onto the perovskite layer and patterned into kagome lattices using an electron-beam lithography process. Another 10.5 pairs of tantalum pentoxide and silicon dioxide were finally deposited using the electron-beam evaporator acting as the top DBR.

### Optical spectroscopy characterizations

The energy-resolved momentum-space and real-space PL mappings were measured using an angle-resolved micro-photoluminescence spectroscopy set-up with a Fourier imaging configuration at room temperature. The emission from the perovskite lattice was collected using a ×50 objective with a numerical aperture (NA = 0.75), then sent to the spectrometer equipped with a grating of 600 lines/mm and a charge-coupled device of 256 × 1024 pixels. By motorized scanning of the optical elements in a Fourier-space imaging configuration, the full dispersion information in *k_x_* and *k_y_* directions was collected. In the linear region, the perovskite lattice was non-resonantly excited using a continuous-wave laser (457 nm) with a pump spot of ∼10 μm. In the non-linear regime, the perovskite lattice was non-resonantly pumped using a pulsed laser (wavelength: 400 nm, pulse duration: 100 fs, repetition rate: 1 kHz) with a pump spot of ∼15 μm. The energy-selected real-space images were measured using a narrow laser-line filter with a linewidth of ∼1 nm on the detection path. The long-range spatial coherence and the first-order correlation were conducted using the MZ interferometer.

### Theoretical calculations

In the strong coupling regime with a finite cavity detuning, we consider the generalized GP equation to describe the dynamics of polaritons in the perovskite microcavity:

(1) \begin{eqnarray*} i\hbar {\rm{\,\,}}\frac{{\partial {\rm{\Psi }}\left( {\boldsymbol{r},t} \right)}}{{\partial t}} &=& \bigg[- \frac{{{\hbar ^2}\nabla _x^2}}{{2{m_x}}} - \frac{{{\hbar ^2}\nabla _y^2}}{{2{m_y}}} + V( \boldsymbol{r})\\ && -\, \frac{{i\gamma }}{2} + g{{\left| {{\rm{\Psi }}( {\boldsymbol{r},t} )} \right|}^2}\\ && -\, i{\alpha _{NL}}{{\left| {{\rm{\Psi }}( {\boldsymbol{r},t} )} \right|}^2} + iP( \boldsymbol{r} )\bigg]\\ &&\times \,{\rm{\,\,\Psi }}\left( {\boldsymbol{r},t} \right), \end{eqnarray*}

where ${m_x}$ and ${m_y}$ represent the effective polariton masses along two perpendicular directions to the crystal axis (the ratio of ${m_x}/{m_y} = \,\,0.7 \pm 0.1$accounts for the anisotropy of the perovskite), an external photonic potential $V( \boldsymbol r )$ is defined within the kagome structure, *g* represents the non-linear polariton–polariton interaction strength and ${\alpha _{NL}}$ denotes the non-linear decay rate of the condensed polaritons. $P( \boldsymbol r )$ is the Gaussian-shaped non-resonant pumping term, noting that the gain inside the kagome lattice is slightly higher than the outside part. In our simulation, we start with the stochastic random initial condition, then read the wave function ${\rm{\Psi }}( {{\boldsymbol r},\,\,t} )$ and the according phase when the system reaches its steady state. Here, the polariton density of the interference image is obtained by the wave function ${\rm{\Psi }}( {r,\,\,t} )$ interfering with a reference plane wave, which can be defined as:

(2) \begin{eqnarray*} I\,\,( {\boldsymbol{r},t}) = {\left| {{\rm{\Psi }}( {\boldsymbol{r},t}) + A{e^{ - {r^2}/{w^2}}}{e^{ik \cdot r}}} \right|^2}\,\,, \end{eqnarray*}

where $A{e^{ - {r^2}/{w^2}}}$ defines a Gaussian-distributed amplitude, in which *A* should be comparable to the magnitude of ${\rm{\Psi }}( {r,\,\,t} )$.

## Supplementary Material

nwac096_Supplemental_File
