# Supplementary material for: Controllable vortex lasing arrays in a geometrically frustrated exciton–polariton lattice at room temperature
Source: Natl Sci Rev. 2022 May 14;10(1):nwac096. doi: 10.1093/nsr/nwac096 (PMC10433738; doi:10.1093/nsr/nwac096)
Supplement: nwac096_Supplemental_File [file nwac096_supplemental_file.docx]

## Supplementary Information for

## Controllable vortex lasing arrays in a geometrically frustrated exciton-polariton lattice at room temperature

Jun Wang^1,4,#,^*, Yutian Peng^2,#^, Huawen Xu^1,#^, Jiangang Feng^1^, Yuqing Huang^1^, Jinqi Wu^1^, Timothy C.H. Liew^1,^*, and Qihua Xiong^2,3,^*

^1^Division of Physics and Applied Physics, School of Physical and Mathematical Sciences, Nanyang Technological University 637371, Singapore.

^2^State Key Laboratory of Low-Dimensional Quantum Physics and Department of Physics, Tsinghua University, Beijing 100084, P.R. China.

^3^Beijing Academy of Quantum Information Sciences, Beijing 100193, P.R. China.

^4^Department of Optical Science and Engineering, and Shanghai Frontiers Science Research Base of Intelligent Optoelectronics and Perception, Fudan University, Shanghai 200433, P.R. China.

^#^These authors contributed equally to this work.

*Corresponding author. Email: [Qihua_xiong@tsinghua.edu.cn](mailto:Qihua_xiong@tsinghua.edu.cn) (Q.X.); [Timothyliew@ntu.edu.sg](mailto:Timothyliew@ntu.edu.sg) (T.C.H.L.); [wangjunfd@fudan.edu.cn](mailto:wangjun13@fudan.edu.cn) (J.W.)

1. **Experimental** **and theoretical dispersions scanned along different directions in the momentum space**

The full band structure is unambiguously demonstrated by tomographic energy-resolved energy-momentum photoluminescence spectra along different cross-sections of momentum space below the threshold, corresponding to dispersions of Fig. 2a-c in the main text. Figures S1a-d show the polaritonic dispersions as a function of $k_{y}$ at $k_{x}=0, \frac{\pi}{3a}, \frac{2\pi}{3a}, \frac{\pi}{a}$, respectively, where *a* of 0.85 μm is the center-center distance of micropillars. The corresponding theoretical simulations of dispersions are performed with a generalized Gross-Pitaevskii (GP) equation, as shown in Fig. S1e-h, in good agreement with the experimental results. Figures S2a-d show the polariton dispersions as a function of $k_{x}$ at $k_{y}=0, \frac{2\pi}{3\sqrt{3}a}, \frac{4\pi}{3\sqrt{3}a}, \frac{2\pi}{\sqrt{3}a}$, respectively. Figures S2e-h show the corresponding theoretical simulations. The experimental and theoretical tomographic dispersions unambiguously demonstrate the appearance of Dirac cones and flat bands in the band structure of the exciton-polaritonic kagome lattice.


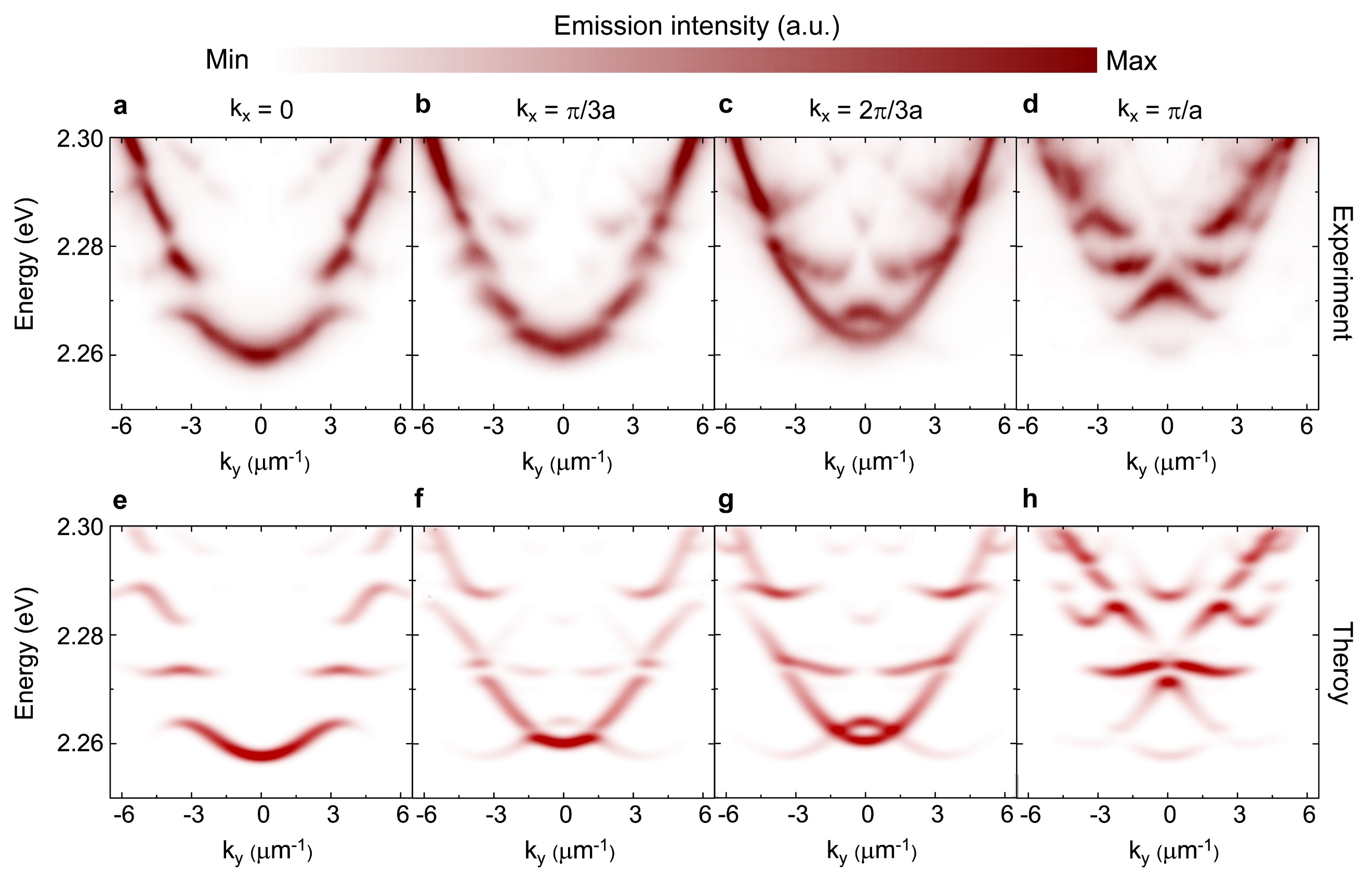


**Figure S1.** Energy-resolved momentum-space polariton dispersions scanned along *k_x_* direction in the linear regime**.** (a-d) Experimental dispersions at $k_{x}=0, \frac{\pi}{3a}, \frac{2\pi}{3a}, \frac{\pi}{a}$. (e-h) Theoretical simulations of (a-d).


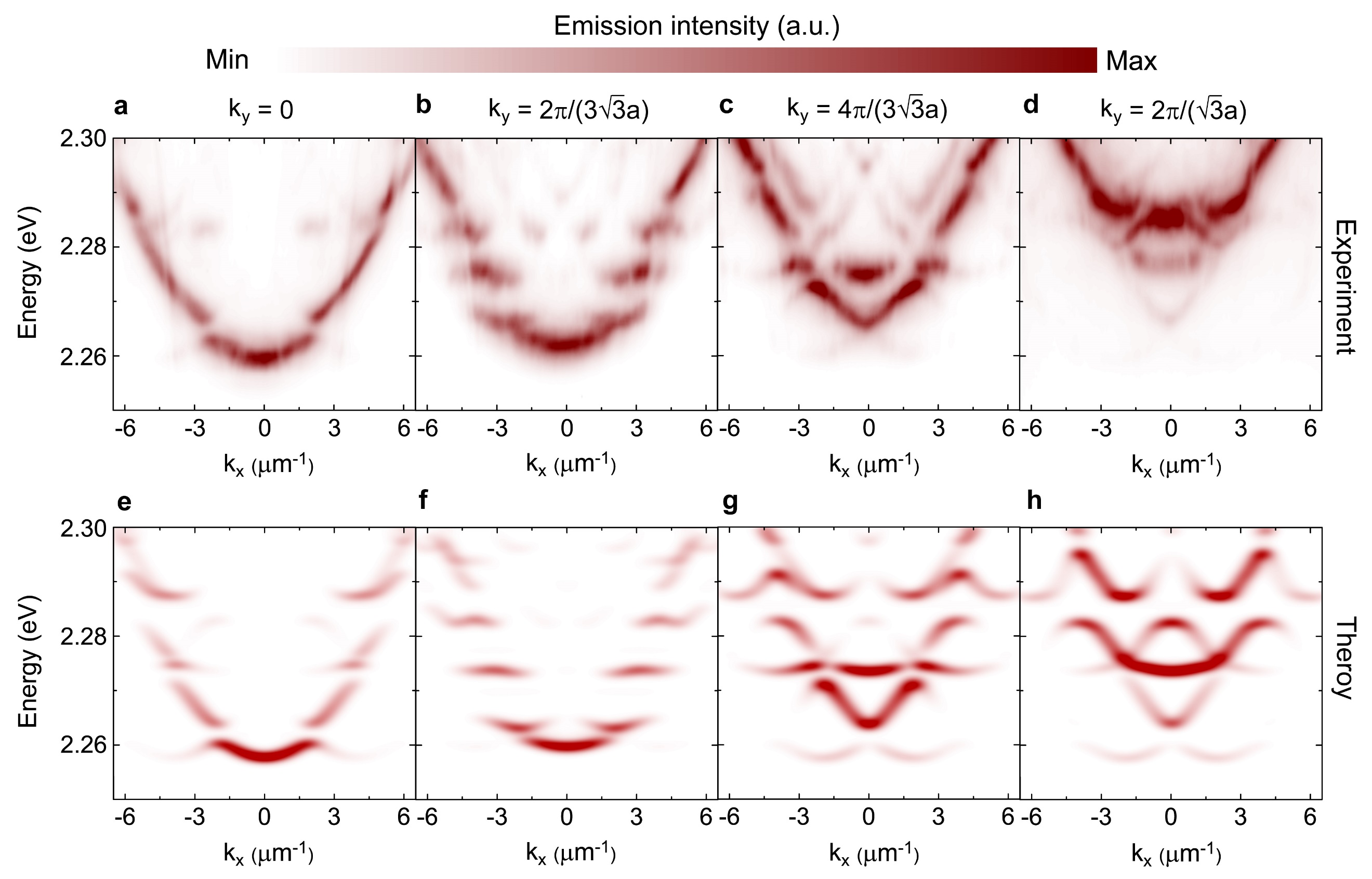


**Figure S2.** Energy-resolved momentum-space polariton dispersions scanned along *k_y_* direction in the linear regime. (a-d) Experimental dispersions at $k_{y}=0, \frac{2\pi}{3\sqrt{3}a}, \frac{4\pi}{3\sqrt{3}a}, \frac{2\pi}{\sqrt{3}a}$. (e-h) Theoretical simulations of (a-d).

1. **Energy-resolved momentum-space images**

In the linear regime of exciton polaritons (below the threshold), the energy-momentum spectrum of Fig. S3a shows the polaritonic dispersion along $K-\Gamma-K'$ direction ($k_{x}=0$). The detuning of this measured sample is slightly different from the sample mentioned in the main text. Figures S3b-d show tomographic momentum-space images at different energies, corresponding to dashed lines in Fig. S3a. Figure S3b clearly shows the first Brillouin zone of a perovskite kagome lattice. On the energies of the *S*-band flat band and *P*-band Dirac points (DPs), their momentum-space images present the hexagonal shape as shown in Fig. S3c and S3d, in agreement with the momentum-space images of their lasing modes above the threshold.


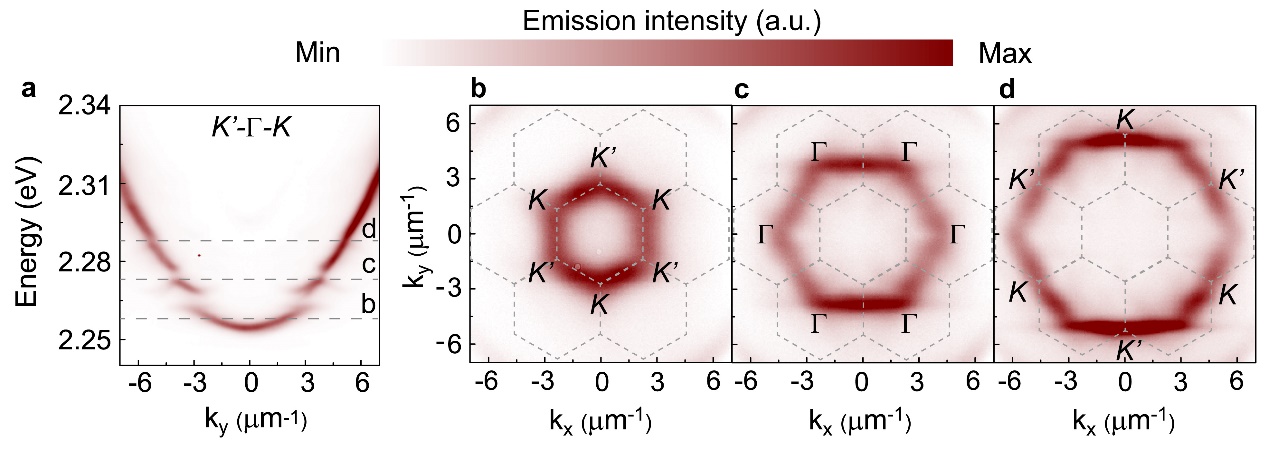


**Figure S3.** Experimental energy-resolved momentum-space images of exciton polaritons in the linear regime. (a) Energy-momentum dispersion of polaritons at $k_{x}=0$. (b-d) Momentum-space images of polaritons at different energies, including *S*-band DPs of (b), *S*-band flat band of (c), the *P*-band state of (d).

1. **Details of interferograms measured from** **Mach-Zehnder interferometer**


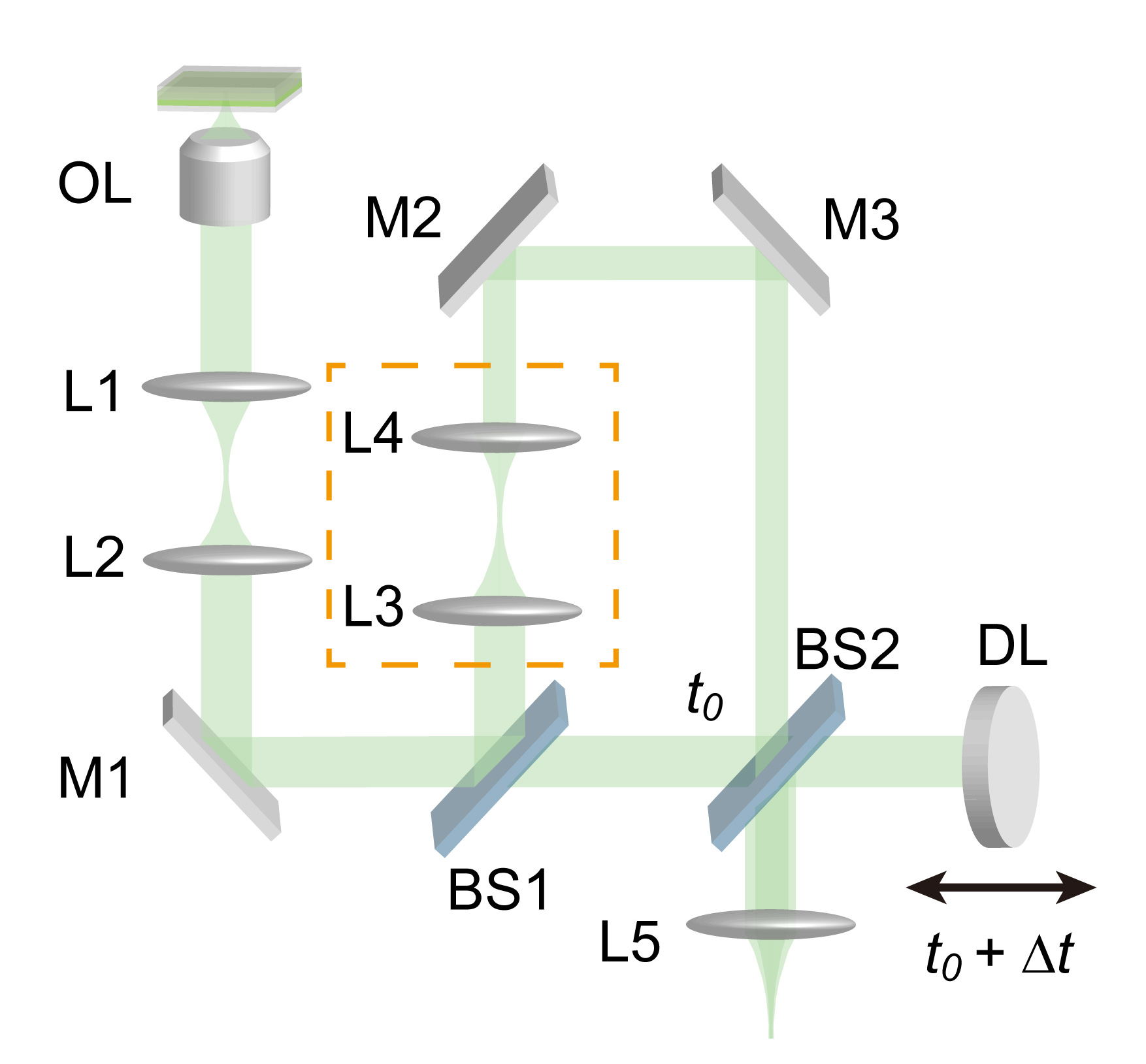


**Figure S4.** Optical setup of the Mach-Zehnder interferometer. DL: delay line, OL: objective, BS: beam splitter, L: lens, M: mirror.

The emergence of long-range spatial coherence of polariton condensates can be proved by a Mach-Zehnder (MZ) interferometer with a telescope in one arm, which allows a magnified emission image as a phase reference, as shown in Fig. S4. Figure S5 comprehensively displays the spatial coherence measurements of the DP state mentioned in Fig. 4 of the main text. Figure S5a shows the real-space image of polariton condensates in the DP state. Figure. S5b clearly exhibits a magnified image of one lobe of the hexagonal condensate pattern, corresponding to the dashed box of Fig. S5a. The magnified lobe can completely cover a kagome plaquette and keep a uniform phase. The corresponding interferogram (Fig. S5c) unambiguously shows discontinuous and staggered fringes, indicating the emergence of a π phase shift in the DP state.


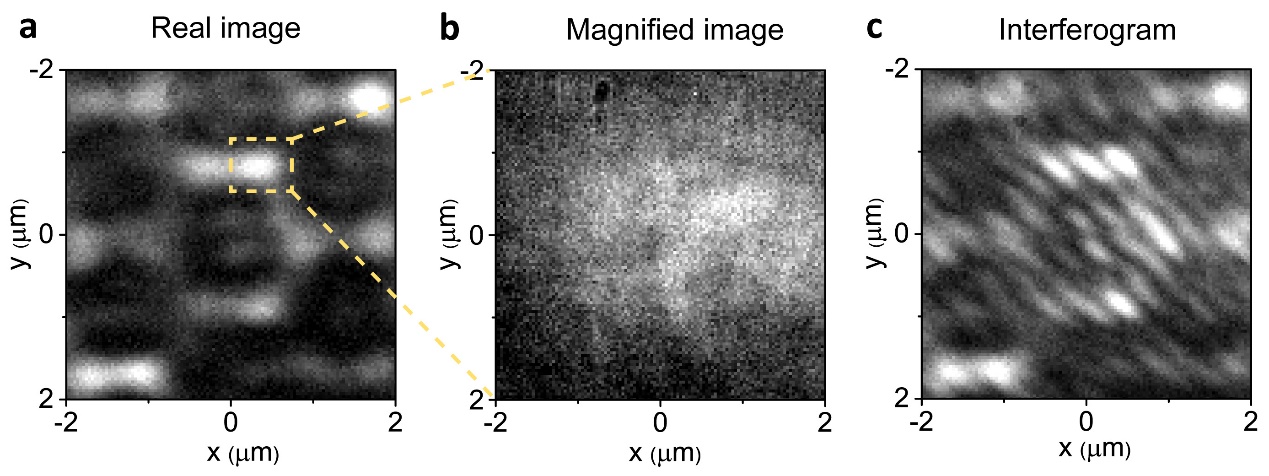


**Figure S5.** Spatial coherence of polariton condensates at the *S*-band DP state in the lattice. (a) Real-space image. (b) Magnified image, corresponding to a lobe in the yellow dashed box of (a). (c) Interferogram of polariton condensates at the *S*-band DP state.


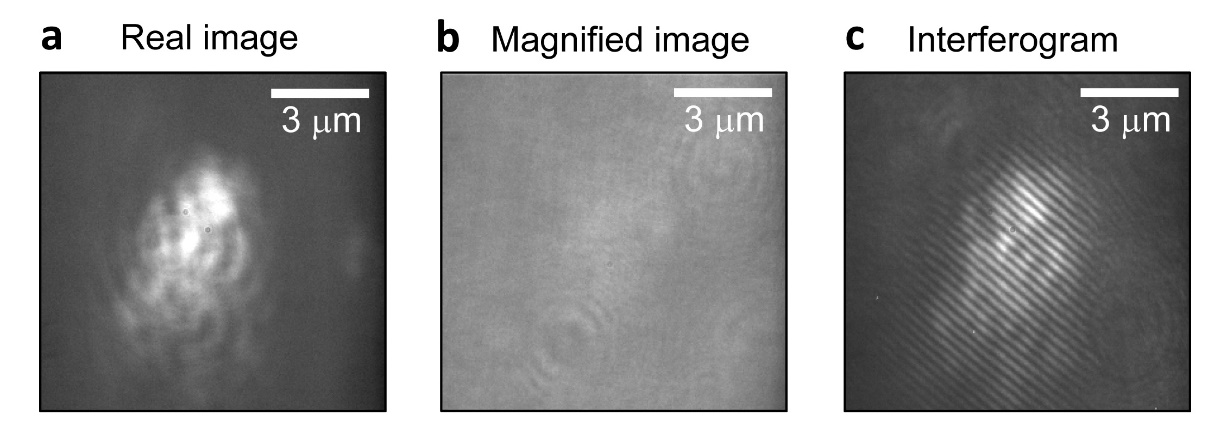


**Figure S6.** Spatial coherence of polariton condensates in the planar microcavity above the threshold. (a) Real-space image. (b) Magnified image of (a). (c) Interferogram. Scale bar: 3 μm.

To further prove the spatial coherence of exciton-polariton condensates in our system, we performed the interference measurement in a planar-microcavity sample above the threshold. A pulse laser with linear polarization was used to non-resonantly excite the planar-microcavity sample, with a laser spot of 6 μm, consequently, polaritons condense at a single mode then lasing. Figure S6a-c shows the real-space image, magnified image and corresponding interference of polariton condensates collected by a spectrometer, respectively. The interferogram shows the parallel stripes throughout the entire laser spot area, which indicates the long-range spatial coherence in a planar microcavity. The size of the interference area is dependent on the spot size. Moreover, the resolution of our MZ interferometer (the period of stripes) approximates 0.3 μm which supports us to distinguish staggered fringes (π phase shifts) and pitchforks (vortices) in the interferogram.

1. **The possible steady-state quantized vortex arrangements**

The odd merging pillars in each unit cell of the frustrated kagome lattice break the parity symmetry, giving rise to the formation of a net polariton current and phase accumulations along each triangular closed loop. In flatband state, due to the non-resonant excitation with the orbital angular momentum of zero, six triangular closed loops of the single kagome plaquette can trap the three vortices ($l=1$) and three antivortices ($l=-1$) above the threshold. In our simulation, by starting with random initial conditions, we performed 200 realizations of the generalized GP equation (see in Methods of main text) and got the wavefunctions at steady states $\Psi\left( \boldsymbol{r},t \right)$. For those realizations that exciton polaritons condense at the flatband, there are three possible quantized vortex arrangements with antiparallel vorticities and net topological charges of zero, which are alternating mode, hybrid antiparallel mode, and half-positive half-negative mode, respectively, as shown in Fig. S7a-c. The energy intervals of such three modes at the flatband are too small to be distinguished by the laser-line filter, approximately 0.3 meV (Fig. S7d), which is much smaller than the linewidth of dispersions. Geometric frustration results in multiple quantized vortex arrangements (topological charge configurations), which is similar to the relative antiferromagnetic spin arrangements in magnetic systems. The appearance probability of hybrid antiparallel mode is dominant than the other two modes (Fig. S7d), because hybrid antiparallel mode possesses more equivalently transformed modes and multiple combinations to extend this configuration over the entire lattice. Moreover, the random initial condition and gain-loss profile of our system tend to underpin the appearance of hybrid antiparallel arrangements, thus this mode is more likely to be observed in the experiments. To further study the variation trend of occurrence probabilities for such three modes, we still performed considerable realizations of the generalized GP equation and calculated their probabilities by tuning the gain-loss ($P, \gamma$) profiles of our system. With the net gain-loss increasing ($W=P-\gamma$), there are some fluctuations in the appearance probabilities of such three modes, but they still maintain a constant variation trend. The appearance probability of hybrid antiparallel mode is dominant than the other two modes in different gain-loss conditions, which is the reason why the flatband exciton-polariton condensates present the hybrid-antiparallel vortex arrangement in the experiment.


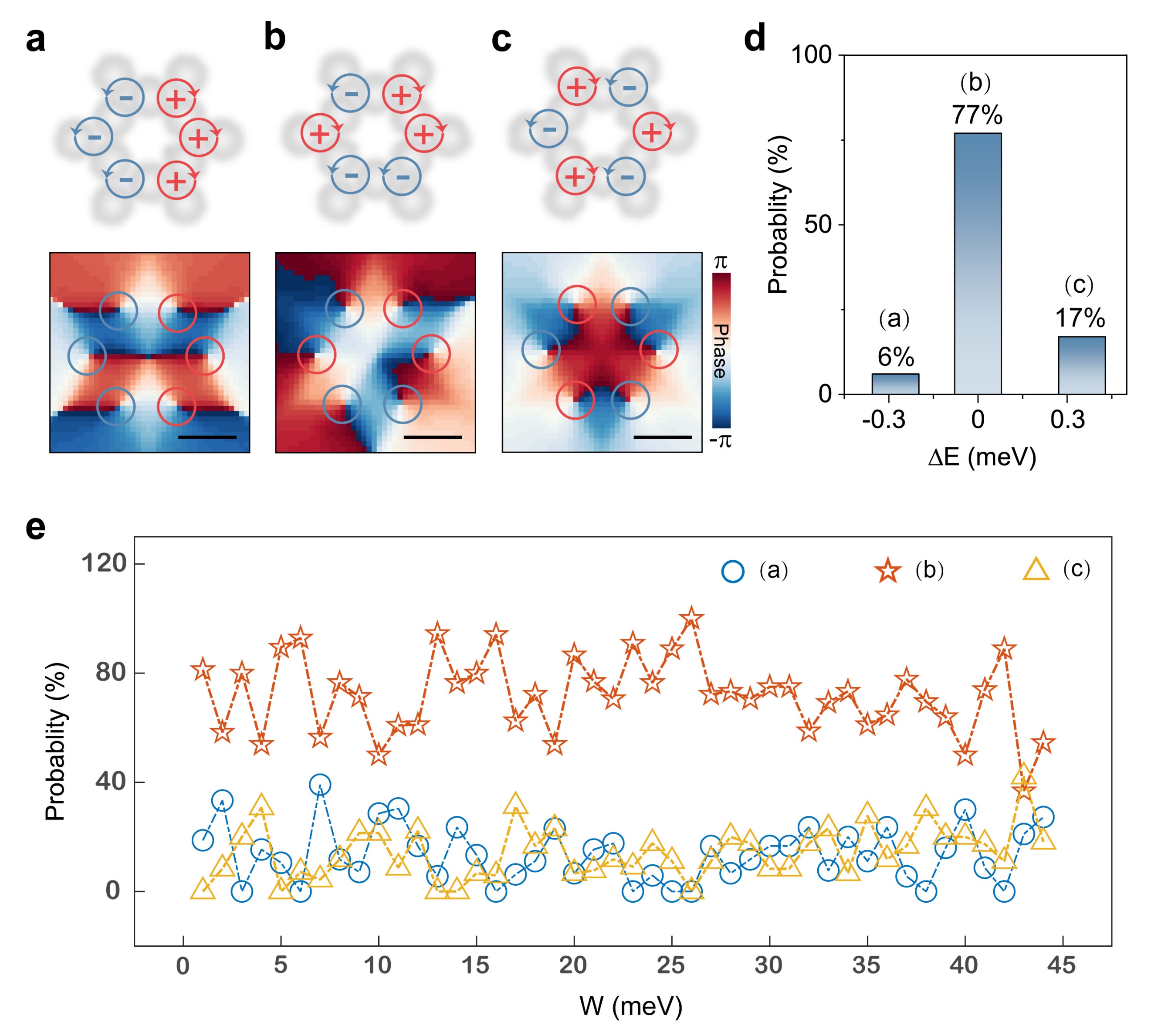


**Figure S7.** Possible steady-state quantized vortex arrangements and their appearance probabilities at the *S*-band flatband state. (a-c), Schematics and phase mappings. (a): half-positive half-negative mode, (b): hybrid antiparallel mode, (c): alternating mode. The scale bar 1 μm. (d) Appearance probabilities of such three configurations. Half-positive half-negative mode: 6%, hybrid antiparallel mode: 77%, alternating mode: 17%. (e) Probabilities of such three configurations with the net gain-loss increasing. The appearance probability of hybrid antiparallel mode is dominant than the other two modes in different gain-loss profiles.

1. **Exciton polaritons in the single micropillar**

In a single micropillar, due to the three dimensions of spatial confinement, the photonic component of the polariton modes will be confined by the potential, then display the discrete energy levels which can be measured from the spatial image of the polariton emission along the diameter of one pillar (Fig. S8a). Figures S8b-c exhibit the real-space images of the polariton emission, where the ground state (*s-*orbital) shows a cylindrical symmetry and the first excited state (*p-*orbital) is two-fold degenerate with antisymmetric orbitals (*p_x_* and *p_y_*) orthogonal to each other.


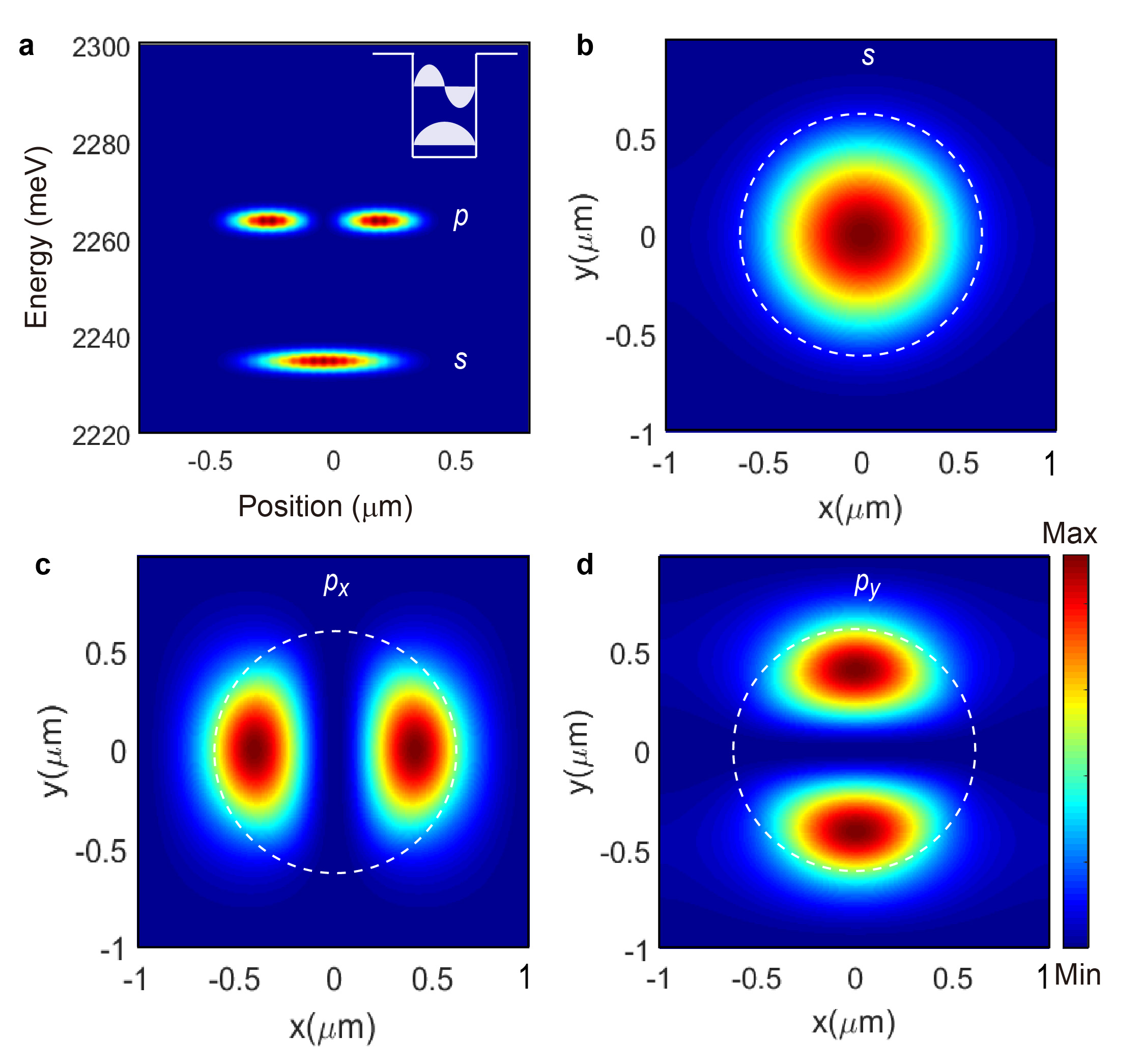


**Figure S8.** (a) Energy-resolved spatial image of a single pillar, showing a non-degenerate *s* state and a twofold-degenerate *p* state. The inset represents the schematic of orbital states in a single pillar. (b) Real-space image of *s*-orbital states in a single pillar, exhibiting a cylindrical symmetry. (c, d) Real-space image of *p*-orbital states in the pillar, exhibiting and the first excited state *p* is twofold degenerate with antisymmetric orbitals (*p_x_* and *p_y_*) orthogonal to each other. The white dashed circles depict the contour of the pillar.
